# A novel *RHD* allele combining partial D (c.455A>C) and weak D (c.1154G>C) variants

**DOI:** 10.1111/trf.18433

**Published:** 2025-09-25

**Authors:** Glenn Ramsey, Kim Hue‐Roye, Gorka Ochoa‐Garay, Johnathon Pugh, Ricardo S. Sumugod, Paul F. Lindholm, Sunitha Vege

**Affiliations:** ^1^ Department of Pathology, Feinberg School of Medicine Northwestern University Chicago Illinois USA; ^2^ Blood Bank, Northwestern Memorial Hospital Chicago Illinois USA; ^3^ Immunohematology and Genomics Laboratory, New York Blood Center Enterprises Rye New York USA

**Keywords:** blood group genomics, immunohematology, Rh blood group

## 1 BACKGROUND

For patients with weak or discrepant RhD phenotypes, *RHD* genotyping is recommended to determine needs for Rh immune globulin (RhIG) prophylaxis and RhD‐negative red blood cell (RBC) transfusion. Patients with weak D types 1, 2, 3, 4.0, and 4.1 can be managed as RhD‐positive, with caveats for type 4.0 in pregnancy; other genotypes are managed as RhD‐negative for uncertain or known risk of anti‐D alloimmunization.^1^ We previously described our hospital‐based and reference laboratory experiences with *RHD* genotyping.^2^, ^3^ The proband here was a 33‐year‐old White woman of southern European ancestry with no history of transfusion presenting at 9 weeks gestation in her first pregnancy.

## 2 BRIEF METHODS

We performed ABO typing, RhD typing (initial direct agglutination (DA) and follow‐up weak D antiglobulin testing), and solid‐phase antibody screens by the automated microplate method (Neo, Werfen, Barcelona, Spain). Manual tube DA typings were obtained five 5 other anti‐D reagents and for RhCcEe antigens. Initial *RHD* genotype was obtained by DNA microarray (RHD BeadChip, Werfen, Barcelona, Spain). To investigate unexpected results, Sanger sequencing of exons 1–10 and flanking regions and site‐directed PCR testing for the hybrid Rhesus box for *RHD* deletion were performed. We subsequently obtained DA neonatal RhD typing.

## 3 RESULTS

The proband's RBCs expressed a weak/negative RhD phenotype in automated testing and 1+ to 3+ agglutination in tube DA tests (Table 1). The RhCcEe phenotype was ccEe. The DNA microarray detected hemi‐ or homozygous (hxm) c.1154G>C and reported weak D type 2 (RHD*01W.02). Inspection of the microarray results also revealed hxm c.455A>C. Sanger sequencing identified those two variants plus c.1154‐31T>C in intron 8. The hybrid Rhesus box was present.

**TABLE 1:** RBC phenotypes of the proband, her neonate, and patients with RHD*01W.02.

Test method/reagents	Proband	Neonate	RHD*01W.02
Automated RhD Neo Series 4/Series 5	Equivocal/Negative		Negative/Negative
Automated weak D Neo Series 4 antiglobulin	4+		3‐4+
Tube RhD Gamma‐clone	3+ mf	4+	1+
Tube RhD^a^ Gamma‐clone, Seraclone	1+		
Tube RhD^a^ BioClone, ALBAclone, *alpha*	2+		
Tube CcEe	C‐c+E+e+		C‐c+E+e+

*Note*: RHD*01W.02: Three cases found by genotyping after 1+ tube typings in confirmatory testing. Neo and Gamma‐clone: Werfen, Barcelona, Spain. Seraclone: Bio‐Rad, Hercules, CA. BioClone: QuidelOrtho, San Diego, CA. ALBAclone and *alpha*: AliveDX, Eysins, Switzerland.

Abbreviation: mf, mixed‐field.

^a^ New York.

## 4 BRIEF SUMMARY

c.455A>C (p.Asn152Thr) is a gene conversion variant from *RHCE* into *RHD* exon 3. In isolation, it comprises the allele RHD*DNT (RHD*38), expressing an apparently normal RhD phenotype but associated with anti‐D.^4^ More often, c.455A>C occurs as one of several variants in alleles of the DIVa phylogenetic cluster. In RHD*01W.02 of the Eurasian cluster, the causative variant c.1154G>C (p.Gly385Ala) is often linked to the intron variant c.1154‐31T>C and in a *cis* arrangement with RHCE*cE. In addition to its amino acid change, c.1154G>C is located at the 5′ end of exon 9 and predicted to disrupt mRNA splicing, accounting for the weak D phenotype.^5^ Our proband's phenotype was similar to three Chicago cases of RHD*01W.02 (Table 1), although this allele's phenotype is variable.^6^ Her RHD*455C, 1154C allele possibly arose from c.455A>C microconversion in a RHD*01W.02 allele. In the Genome Aggregation Database, none of 111,705 subjects had both c.455C and c.1154C (v2.1.1, variant co‐occurrence). This novel allele was submitted to GenBank (accession PQ858709). c.455A>C was previously observed in *cis* with weak D type 1 variant c.809T>G (p.Val270Gly) in the RHD*62 allele (phenotype unknown) (GenBank LN554881).

This case illustrates the importance of reviewing genotyping tests for unexpected findings. Because of published anti‐D risk with c.455A>C, we overrode the RHD*01W.02 microarray call and recommended Rh immunoprophylaxis and RhD‐negative RBC transfusions if needed. The patient received RhIG at 21 weeks for bleeding (placenta previa), 28 weeks, and postpartum after delivering an RhD‐positive newborn (not genotyped) at 37 weeks. RBC antibody screens were negative at 9, 28, and 37 weeks gestation. Nondetection of antenatal RhIG in antibody screens at delivery in women with partial *RHD* variants has raised concern for maternal RhIG adsorption and immunoprophylaxis inadequacy.^7^

## CONFLICT OF INTEREST STATEMENT

Glenn Ramsey is a consultant at Werfen.

## References

[1] Flegel WA , Denomme GA , Queenan JT , Johnson ST , Keller MA , Westhoff CM , et al. It's time to phase out “serologic weak D phenotype” and resolve D types with *RHD* genotyping including weak D type 4. Transfusion. 2020;60:855–859. 32163599 10.1111/trf.15741 PMC9121350

[2] Barriteau CM , Lindholm PF , Hartman K , Sumugod RD , Ramsey G . *RHD* genotyping to resolve weak and discrepant RhD patient phenotypes. Transfusion. 2022;62:2194–2199. 36218305 10.1111/trf.17145 PMC9828470

[3] Vege S , Sprogøe U , Lomas‐Francis C , Jakobsen MA , Antonsen B , Aeschlimann J , et al. Impact of *RHD* genotyping on transfusion practice in Denmark and the United States and identification of novel *RHD* alleles. Transfusion. 2021;61:256–265. 32975828 10.1111/trf.16100

[4] Lee GH , Kim H , Hur M , So KA , Shin DW , Hong YJ , et al. RHD*DNT (RHD*38) showing D‐positive reactivity on Rhesus D typing and forming anti‐D antibody. Ann Lab Med. 2023;43:524–527. 37080757 10.3343/alm.2023.43.5.524 PMC10151267

[5] Raud L , Ka C , Gourlaouen I , Callebaut I , Férec C , le Gac G , et al. Functional analysis of novel *RHD* variants: splicing disruption is likely to be a common mechanism of variant D phenotype. Transfusion. 2019;59:1367–1375. 30811032 10.1111/trf.15210

[6] Theiler C , Lomas‐Francis C , Vege S , Chevrier MC , Leiva‐Torres GA , Keller MA , et al. Weak and partial D phenotyping: a comparison study between molecular and serologic results. Immunohematology. 2024;40:159–165. 39740012 10.2478/immunohematology-2024-022

[7] Toly‐Ndour C , Floch A , Huguet‐Jacquot S , Smith GCS , Spiegelhalter DJ , Squires H , et al. Rh disease prevention for women with partial D: is routine antenatal anti‐D prophylaxis useful? Vox Sang. 2023;118(S1):91–92.

[trf18433-bib-0008] https://www.isbtweb.org/working‐parties/red‐cell‐immunogenetics‐and‐blood‐group‐terminology. Accessed 6 Jun 2025

[trf18433-bib-0009] https://gnomad.broadinstitute.org/. Genome Aggregation Database (gnomAD). Accessed 6 Jun 2025

